# Management of decontamination in chemical accidents: a laboratory model

**DOI:** 10.1038/s41598-023-35248-8

**Published:** 2023-05-24

**Authors:** Tobias Hundhammer, Regina Lindner, Leopold Caccia, Hannes Langbehn, Walter Petermichl, Michael Dittmar, Michael Gruber

**Affiliations:** Department of Anesthesiology, University Hospital Regensburg, Franz-Josef-Strauß-Allee11, 93042 Regensburg, Germany

**Keywords:** Medical and clinical diagnostics, Chemical safety, Disease prevention, Skin diseases, Analytical chemistry

## Abstract

Rapid and efficient decontamination of the skin is a major task for emergency rescue services in the event of a chemical accident involving humans. While rinsing the skin with water (and soap) has been the standard procedure, some skepticism has developed in recent years regarding the situational suitability of this method. The efficacy of three different decontamination materials/techniques (Easyderm® cleaning cloth, water-soaked all-purpose sponge, rinsing with water) in removing Capsaicin, Bromadiolone, Paraquat and 2,2′-dichlorodiethylether (DCEE) from porcine skin was compared. Different cleaning motions (wiping, twisting, pressing) with the Easyderm® were evaluated for their effectiveness in removing Capsaicin from porcine skin. Finally, the impact of different exposure times of the skin to Capsaicin on the decontamination process were investigated. Contaminant recovery rates (CRRs) were analysed in the skin and in each decontamination material using high-performance-liquid-chromatography (HPLC; used for Capsaicin, Bromadiolone, Paraquat) or gas chromatography (GC; used for DCEE). Wiping the skin with the amphiphilic Easyderm® was most effective for decontamination of Capsaicin and DCEE, while the water rinsing method gave the best results for removing Paraquat and Bromadiolone. Both wiping with the Easyderm® and rotating the Easyderm® were significantly more effective in cleaning Capsaicin-contaminated skin than pressing the Easyderm® on the contamination area alone. Prolonged exposure times of the porcine skin to Capsaicin were associated with a decrease in efficacy of the following decontamination. Emergency rescue services should have materials available that can remove both hydrophilic and hydrophobic substances from skin. Since not all of our results for comparing different decontamination materials were as distinct as we expected, there are likely several other factors determining the efficacy of skin decontamination in some cases. Time is key; therefore, first responders should try to begin the decontamination process as soon as possible after arriving at the scene.

## Introduction

Accidents involving chemical substances can occur in a variety of circumstances, be it chemical industry, agriculture, laboratories, private households, or even nature catastrophes and terrorist attacks. The World Health Organisation (WHO) estimates that chemical accidents have caused 65,000 deaths worldwide between 2009 and 2018^1^. While the majority of intoxications probably occurs via inhalation, cases of dermal contamination must not be neglected. The transportation of chemicals poses a particular risk, as accidents on transportation routes may affect a particularly large number of people. In Germany, 137 million tons of chemicals were transported by road and 25 million tons were transported by rail in 2020^2^. Despite extensive safety precautions, accidents involving the transportation of hazardous materials still cannot be entirely prevented. In 2021, the U.S. Department of Transportation reported a total of approximately 23,000 accidents involving chemical substances, 95% of which occurred on highways^3^.

The European Chemicals Agency (ECHA) currently lists about 100,000 registered chemical substances^4^. While some of those are considered non-hazardous for humans, others can cause serious health issues through contamination via inhalation, oral ingestion, or skin-contact.

It is therefore of great importance that emergency medical services (EMS) have access to rapid and effective decontamination methods in the event of chemical accidents involving humans.

In our laboratory model, contamination with the following four chemical substances was simulated:

2,2′-dichlorodiethylether (DCEE) is a marginally volatile, clear or yellowish liquid. It is moderately lipophilic and only slightly soluble in water. DCEE is mainly used as a solvent for resins, lipids and rubber, but also as a pesticide^5^. DCEE is an oxygen analogue to sulphur mustard (SM), which is a chemical warfare agent. Because it has a similar structure but lacks the dangerous blistering effect of sulphur mustard, DCEE has been used as a more manageable surrogate for SM in several studies in the past^6,7^.

Oleoresin Capsicum (OC) is the main irritant of conventional pepper spray. Its pharmacologically active substances are capsaicinoids, which are produced in the secondary metabolism of plants of the genus Capsicum (e.g., chili pepper or bell pepper). Out of the 5 naturally occurring capsaicinoids, Capsaicin and Dihydrocapsaicin are the two most potent and abundant^8^. Capsaicin is a crystalloid, very lipophilic and virtually water-insoluble substance^9^, which is resorbed into the epidermis and dermis after dermal application. Skin contact with Capsaicin may cause local irritation concomitant with pain, reddening and swelling of the contaminated area^10^.

Paraquat is a very hydrophilic quaternary ammonium compound formed by dissociation of the salts Paraquat dichloride (PDC) or Paraquat dimethyl sulphate (PDM) in aqueous solution^11^. Although its use is prohibited in the European Union (EU)^12^, Paraquat is still widely utilized as a contact herbicide in many other countries, including the United States (US). Dermal contamination with Paraquat can cause skin irritation with blistering and ulceration^13^. Furthermore, percutaneous absorption of Paraquat can also lead to toxic systemic effects, such as renal and respiratory failure^14^.

Bromadiolone is a compound consisting of four stereoisomers, which forms an odourless, white powder at room temperature. It is practically insoluble in water, but can be dissolved in dimethylformamide^15^. Bromadiolone has been approved as a rodenticide in the EU since 2009^16^. Belonging to a category of rodenticides called “super-warfarins”, Bromadiolone inhibits blood clotting in rodents, causing them to decease through internal bleeding within 3–7 days after ingestion^17^. Haematuria, haematochezia, epistaxis and ecchymosis along with prolonged prothrombin time (PT) and low levels of vitamin K-dependent coagulation factors are the common symptoms of super-warfarin intoxication^18,19^. However, intracerebellar and subarachnoid haemorrhage have also been reported^20,21^. While in most cases the poisoning occurred through accidental or suicidal oral ingestion, there is at least one case report of super-warfarin poisoning through dermal exposition^22^, with this patient showing symptoms similar to oral ingestion.

Our study aims to re-evaluate the existing methods for decontamination of skin from potentially hazardous chemicals. A laboratory model was established in which the decontamination of porcine skin with different cleaning methods could be simulated under standardized conditions. Two hydrophobic (Bromadiolone, Capsaicin), one slightly hydrophilic (DCEE) and one very hydrophilic (Paraquat) chemical substance were selected as representatives of potential substances involved in chemical accidents. Three different decontamination techniques were compared: rinsing with water, wiping with a water-soaked sponge (Spontex® all-purpose sponge, MAPA GmBH, Zeven, Germany) and wiping with Easyderm® cleaning tissue (Medi GmbH & Co. KG, Bayreuth, Germany), which will be called “Easyderm®” in the following. After the decontamination process, the substance concentrations remaining on the skin as well as those absorbed into the cleaning material were quantified by gas chromatography (GC) or high-performance-liquid-chromatography (HPLC).

Using this laboratory model, the following questions will be assessed in this study:

(I) Do the chemical properties, especially the water solubility, of the contaminating agents determine which decontamination method is most suitable for removing the substance from the skin?
(II) When using the Easyderm®, is there a significant difference between cleaning techniques/motions regarding the efficacy of substance removal?
(III) Does the time of exposure of the skin to the contaminant affect the efficiency of subsequent decontamination?

## Materials and methods

### Porcine skin

The non-scalded porcine skin was supplied by a local butcher. To separate epidermis, dermis and upper subcutis from the lower structures, the skin was cut into a layer of 3–4 mm. Until usage, the skin was stored at − 20 °C under airtight conditions.

For each experiment, a strip of frozen skin was placed on a polystyrene plate wrapped in aluminum foil and plastic wrap and secured with fixing pins. The skin was thawed for 30 min at room temperature. Condensed water was dabbed away with a paper towel.

**Figure 1:**
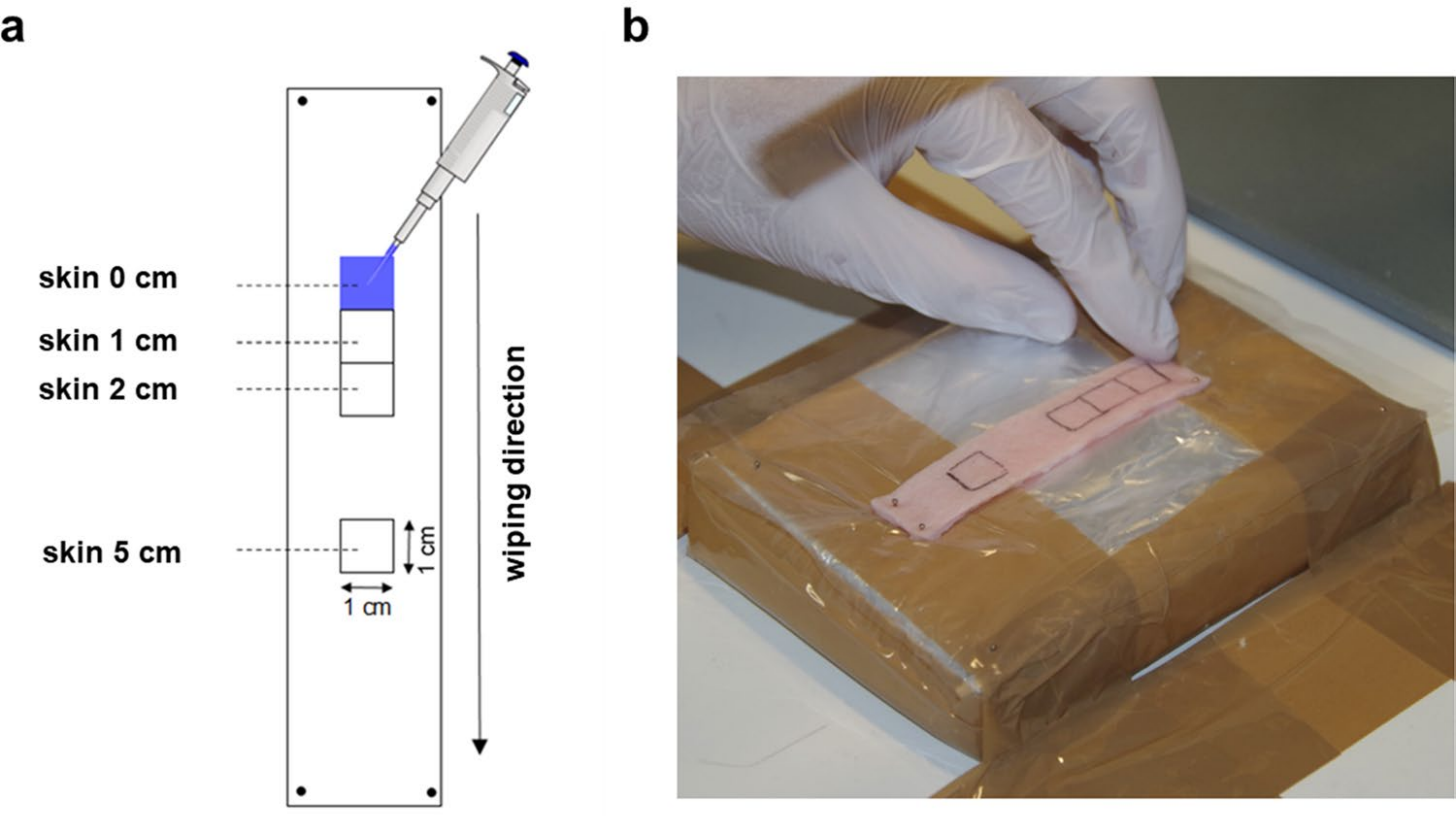
Illustration of the porcine skin strip used for the decontamination model. (**a**) Scheme of the strip in which four squares of 1 cm^2^ are marked. “Skin 0 cm” served as the application area for the contaminant and is marked with blue coloring and a pipette. (**b**) Image of the skin model attached to the polystyrene plate with pins.

Four squares of 1 cm^2^ each were marked on the skin strip (Fig. 1): The first square was designated as the application area for the contaminant. Three further squares were marked at intervals of 1, 2 and 5 cm in wiping direction, downstream of the application area.

### Contamination procedure

Four substances were selected as contaminants for this study: 2,2′-dichlorodiethylether (DCEE), Paraquat, Bromadiolone and Capsaicin. Depending on their chemical properties, they were dissolved in different solvents (Table 1).

**Table 1:** Constitution of the four contamination agents used in this study. *Capsaicin natural, as stated by the manufacturer, consists of 65% Capsaicin and 35% Dihydrocapsaicin.

Active agent	Solvent	Concentration of active agent (mg/mL)	Volume of contaminant applied to pigskin (µL)
Bromadiolone	Dimethylformamide/Ethanol (10/90)	1	10
Capsaicin natural*	Isopropanol (100%)	20	5
2,2’-Dichlorodiethylether (DCEE)	Distilled water	1	5
Paraquat	Distilled water	1	10

For contamination, 5 or 10 µL of the chemical to be tested were applied to the application area (“skin 0 cm”) in a meandering line. For comparison of different decontamination materials as well as different cleaning techniques with the Easyderm®, exposure time between contaminant and skin was 10 min. To further investigate the influence of exposure time on decontamination efficacy, the pigskin was exposed to Capsaicin for either 1, 10, 30 or 60 min.

### Decontamination procedures

#### Decontamination with water

A continuous water flow was generated using a peristaltic pump (Minipuls® 3, Gilson®, Middleton, USA). The contaminated skin was rinsed for 30 s under a homogenous flow of in total 12 mL of tap water (21.5 °C). The eluate was collected in a centrifugation tube for further analysis.

#### Decontamination with EasyDerm®

**Figure 2:**
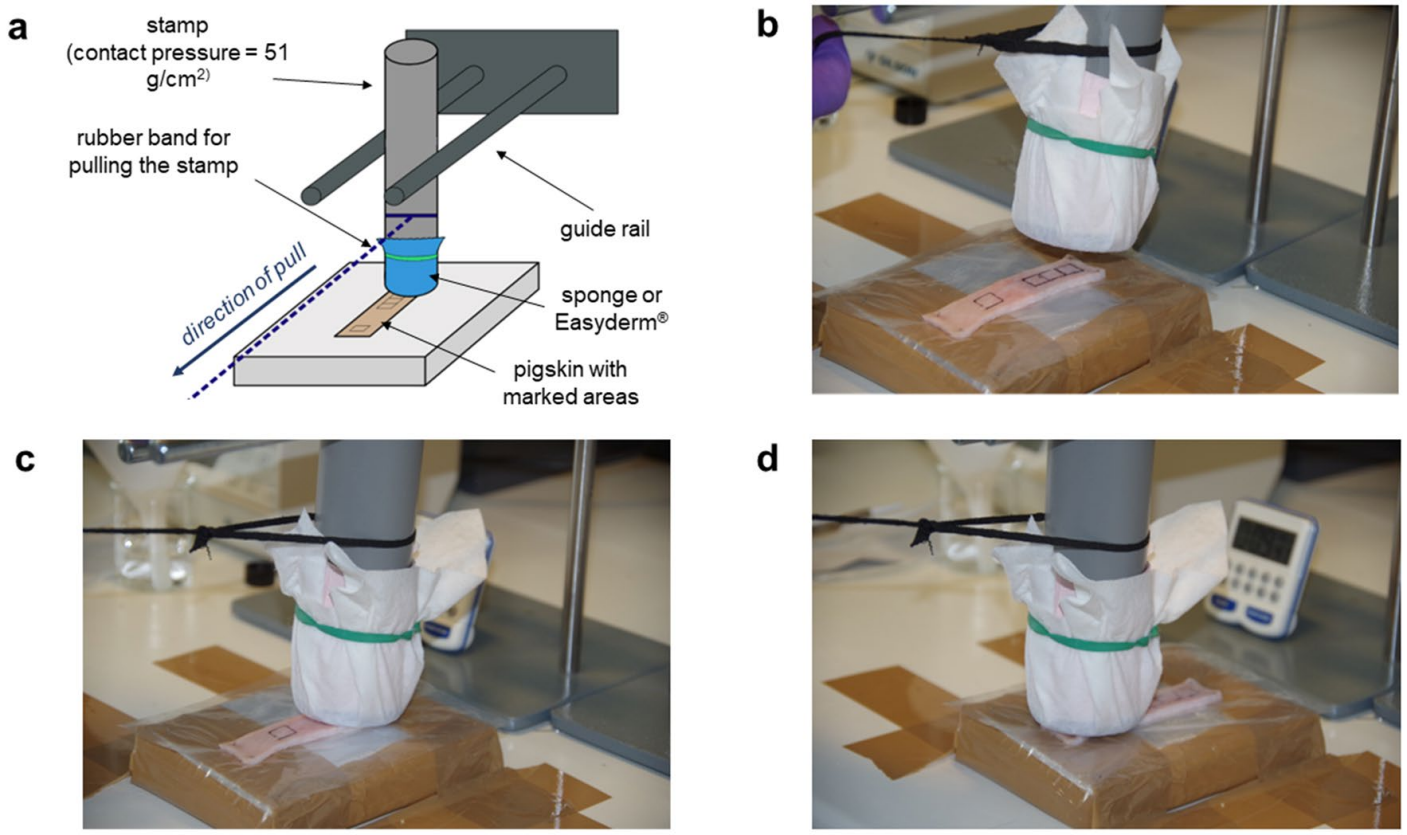
Decontamination of skin with EasyDerm® or Spontex® all-purpose sponge. (**a**) Schematic image of the experimental setup. (**b**)–(**d**) unidirectional wiping away from the decontamination site (“skin 0 cm”).

To provide a constant contact pressure (51 g/cm^2^) between the Easyderm® and the skin, the 10 × 10 cm square of Easyderm® was attached to a stamp filled with small metal beads (Fig. 2). Three different decontamination methods were performed:

- “Wiping”: Unidirectional wiping away from the contamination spot; 4 repetitions
- “Twisting”: Putting the stamp on the contamination spot, then twisting by 360°; 4 repetitions
- “Pressing”: Impression of the stamp on the contamination spot without motion for 1 min

After decontamination, a pre-marked area of 19.6 cm^2^ was excised from the Easyderm® for further analysis.

#### Decontamination with Spontex® all-purpose sponge

Circles with a diameter of 2.5 cm (circular area = 4.9 cm^2^) and a thickness of approximately 1 cm were cut out of Spontex® all-purpose sponge (MAPA GmBH), soaked with 1 mL of tap water (21.5 °C) and attached to the stamp head. For decontamination, the “wiping” technique was used as described for the EasyDerm®.

### Sample preparation

To examine the efficacy of each decontamination technique, the pre-marked skin areas (“skin 0 cm”, “skin 1 cm”, “skin 2 cm”, “skin 5 cm”) and the decontamination materials (water, Easyderm®, sponge) were analyzed regarding the residues of the respective contaminants. For analysis of the skin, the pre-marked areas were cut out with a scalpel. Depending on the following analysis method (GC or HPLC), the sample preparation for each contaminant differed.

#### Preparation of Capsaicin samples

The skin samples were each placed in 20 mL glass vials and mixed with 2 mL of isopropanol and 50 µL Phenacetin (2 mg/mL) dissolved in isopropanol, which served as an internal standard. The sponge and Easyderm® samples were each placed in 20 mL glass vials and mixed with 6 mL of isopropanol and 50 µL of Phenacetin. 9 mL of the water eluate were mixed with 50 µL of Phenacetin inside a glass vial. The vials were placed into an ultrasonic bath (Sonorex super RK 510H, Berlin, Germany) for 10 min at room temperature.

1.5 mL of each sample were transferred to conical centrifugation tubes and completely evaporated at 40 °C under constant N_2_-flow. The residues were resuspended in 100 µL of isopropanol and centrifuged at 16,000 g for 3 min. Finally, the supernatants were transferred into micro vials for analysis by HPLC.

#### Preparation of Bromadiolone samples

The skin slices, Easyderm® and sponge samples were placed in 10 mL headspace vials and mixed with 5 mL ethanol and 10 µL of ethanol-dissolved internal standard Warfarin (1 mg/mL). The vials were shaken for 30 s and then placed in an ultrasonic bath at room temperature for 10 min. The samples were evaporated at 70 °C under constant N_2_-flow. After the residues were resuspended in 200 µL ethanol and centrifuged at 3000 g for 10 min, 50 µL of each supernatant were extracted and analyzed by HPLC.

The sample containing 12 mL water eluate was mixed with 10 µL of warfarin and then evaporated at 90 °C under constant N_2_-flow, resuspended in 200 µL of ethanol and analyzed via HPLC.

#### Preparation of Paraquat samples

Skin, Easyderm® and sponge samples were each placed in 10 mL headspace vials and mixed with 5 mL of water and 20 µL of Diethylparaquat (DEP; 1 mg/mL), which served as an internal standard. The mixture was shaken for 30 s and then placed in an ultrasonic bath for 10 min at room temperature. 1 mL of the suspension was transferred into a centrifugation tube and centrifuged at 16,000 g for 7 min. Thereafter, 200 µL from the lower phase of the suspension were extracted and analyzed by HPLC.

The sample containing 12 mL of the water eluate was mixed with 20 µL of DEP. The mixture was shaken, then 2 mL of it were evaporated at 90 °C under constant N_2_-flow. The residue was resuspended in 200 µL of water, transferred into a headspace vial and analyzed by HPLC.

#### Preparation of DCEE samples

The skin, Easyderm®, and sponge samples as well as 1 mL of the water eluate were each placed in 10 mL headspace vials and mixed with 1 mL of distilled water, 100 µL of methoxyethanol and 10 µL of halothane dissolved in methoxyethanol (6.96 mg/mL), which served as an internal standard. All samples were placed in an ultrasonic bath for 10 min at room temperature. Afterwards, the samples were equilibrated by placing the glass vials for 35 min in a 40 °C water bath (SWB 25, Thermo Haake, Karlsruhe, Deutschland), which was shaking at a frequency of 40 min^−1^. Thereupon, the samples could be analyzed by GC.

### Analysis

#### Analysis via HPLC

The HPLC conditions under which the analyses were performed are listed in Table 2. The precolumn for each substance was C18 ODS (Phenomenex, Aschaffenburg, Germany). The separation columns were C18 (ZORBAX Eclipse XDB-C18 4.6 × 50 mm, 1.8 µm, Agilent Technologies, Santa Clara, USA) for Capsaicin/Dihydrocapsaicin and Bromadiolone, and C8 (ZORBAX Eclipse XDB-C8, 4.6 × 75 mm, 1.8 µm, Agilent Technologies) for Paraquat.

**Table 2:** HPLC conditions for analysis Capsaicin, Bromadiolone and Paraquat concentrations.

Substance	Internal standard	Separation column	Liquid phase buffers	Injection volume	Detection wavelength	Flow rate; Temperature
Capsaicin/Dihydrocapsaicin	Phenacetin	C18	A: Acetonitril/10 µM phosphate buffer (10:90, v/v)	2 µL	280 nm	2 mL/min; 38 °C
			B: Acetonitril/10 µM phosphate buffer (90:10, v/v)			
Bromadiolone	Warfarin	C18	A: Sodium octanesulfonate in H_2_0 (100 µM)	10 µL	260 nm	1.5 mL/min; 36 °C
			B: Acetonitril/H_2_O (90:10, v/v)			
Paraquat	DEP	C8	A: Sodium octanesulfonate in H_2_0 (10 µM)	3 µL	254 nm	2.5 mL/min; 36 °C
			B: Acetonitril/H_2_O (28:72, v/v)			

The following gradients were driven for the respective substances:

- Capsaicin/Dihydrocapsaicin: Starting at 15%, the fraction of buffer B was linearly increased for 3 min up to 40%. This composition of the liquid phase was then maintained for 5 min and then decreased by 25% per minute until the initial condition was reached again. The overall run-time was 12 min.
- Bromadiolone: Buffer A started at 50% for the first 1.5 min, which was then decreased to 40% within the next minute, where it remained for up to 7.5 min. Thereafter, buffer A was increased to 50% again within the following half minute. The overall run-time was 8 min.
- Paraquat: An isocratic gradient of 20% buffer B was run for 4.5 min.

#### Analysis via GC-FID

After 35 min of equilibration time, 2 mL of the gas phase were withdrawn and directly on column injected. A polyphenylmethylsiloxan capillary column (30 m × 0.53 mm × 3 µm; DB-624, J & W Scientific, Folsom, USA) was used for chromatographic separation. A temperature gradient was run: it started at 50 °C for 1 min and had 4 min to rise to 150 °C. After 3 min it dropped back to 50 °C again. The gas flow was: 1.1 bar H_2_, 1.2 bar synthetic air at the flame ionization detector (FID) and 1.2 bar Helium, which served as the carrier gas on the column.

### Statistical analysis

For statistical analysis, SPSS® statistics software program (version 28.0.0.0, IBM, Armonk, USA) was used. Results were tested for Gaussian distribution using the Kolmogorov–Smirnov test. Results with normal distribution were analysed via one-way ANOVA, Levene’s test was used to determine homogeneity of variance. Bonferroni’s test (homogeneity) or Games-Howell’s test (no homogeneity) were used for post-hoc analysis.

If the data did not show normal distribution, non-parametric Kruskal–Wallis’s test followed by Bonferroni correction were performed.

A linear regression analysis and subsequent Cohen’s *f*^2^ test were performed to investigate the correlation between the skin’s time of exposure to the contaminant and the contaminant recovery rate after decontamination.

## Results

### Decontamination efficacy of Easyderm®, sponge and water rinsing methods

Three decontamination methods were examined in this part of the study: Rinsing with water, wiping with Spontex® all-purpose sponge, and wiping with EasyDerm®. The number of replicates for each method and substance is shown in Table 3.

**Table 3:** Number of replicates for each substance and decontamination method when comparing Easyderm® wiping, sponge wiping and water rinsing methods.

Substance	Decontamination method	Number of replicates
Capsaicin/Dihydrocapsaicin	Easyderm® wiping	n = 20
	Sponge wiping	n = 20
	Water rinsing	n = 20
Bromadiolone	Easyderm® wiping	n = 22
	Sponge wiping	n = 19
	Water rinsing	n = 41
DCEE	Easyderm® wiping	n = 20
	Sponge wiping	n = 20
	Water rinsing	n = 20
Paraquat	Easyderm® wiping	n = 20
	Sponge wiping	n = 21
	Water rinsing	n = 41

**Figure 3:**
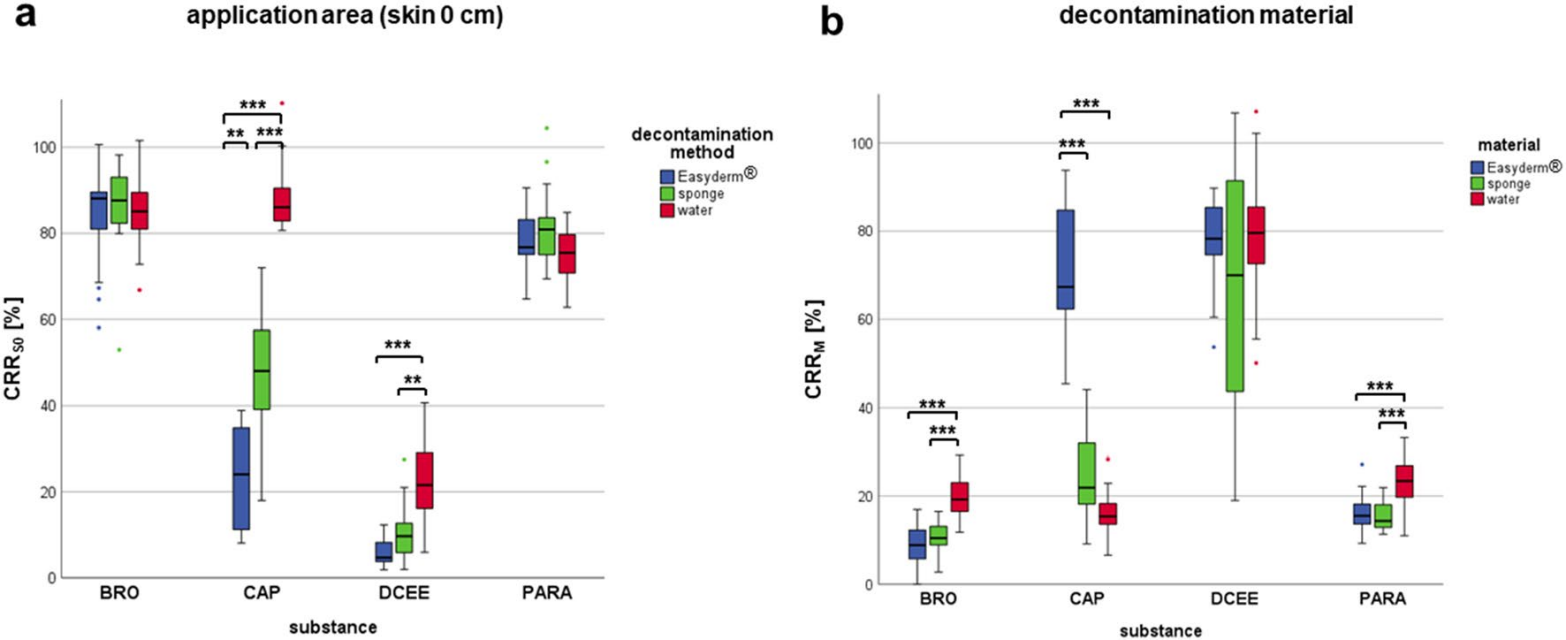
Recovery rates of Bromadiolone (BRO), Capsaicin (CAP), DCEE and Paraquat (PARA) in the application area (**a**) and in all decontamination materials (**b**). Significant differences are marked with ** (*p* < 0.01) and *** (*p* < 0.001). Non-significant differences are not indicated.

For each contaminating substance, the contaminant recovery rate (CRR) was analysed in the application area (skin 0 cm), the three skin areas downstream of the application area (skin 1–5 cm) and in the respective decontamination material (Fig. 3).While a high contaminant recovery rate in the decontamination material (“CRR_M_”) corresponded to more effective decontamination, high contaminant recovery rates in the skin samples (“CRR_S0_” for application area, “CRR_S1_” for skin 1–5 cm) corresponded to ineffective decontamination.

When contamination was performed with hydrophobic Bromadiolone, mean CRR_S0_ values after cleaning ranged between 83.8 and 86.7% and did not differ significantly between the decontamination methods (Fig. 3a). However, the water rinsing method resulted in 19.6% of the contaminant being detected in the eluted water as opposed to only 10.9% in the sponge and 9.8% in the Easyderm® (Fig. 3b). This difference was statistically significant (*p* < 0.001 for both sponge and Easyderm®).

Contamination with hydrophobic Capsaicin led to different results. While the mean CRR_S0_ was still 88.2% after decontamination with water, cleaning the skin with the sponge (47.4%; *p* < 0.001) or Easyderm® (22.7%; *p* < 0.001) resulted in a significantly more effective decontamination. The difference between Easyderm® and sponge was statistically significant as well (*p* < 0.01). Examination of the CRR_M_ in the respective cleaning materials confirmed this observation: only a low rate of Capsaicin and Dihydrocapsaicin could be detected in both the eluted water (16.5%) and the sponge (24.0%). However, most of the Capsaicin once applied could be found in the Easyderm® after decontamination (70.2%), which was significantly more than in the eluted water (*p* < 0.001) and in the sponge (*p* < 0.001). In summary, rinsing with water and wiping with the sponge left most of the Capsaicin on the skin, while wiping with the Easyderm® removed about 70% of the contaminant.

Examination of the moderately hydrophilic contaminant DCEE showed that all three cleaning methods achieved good decontamination results, as contaminant recovery in each cleaning matrix (sponge, Easyderm®, water) was above 67.2% but did not differ significantly. However, decontamination by water rinsing led to significantly more (22.8%) DCEE remaining on the application area than the sponge (10.3%; *p* < 0.01) or Easyderm® (6.1%; *p* < 0.001) methods. There were no significant differences between sponge and Easyderm® at “skin 0 cm”.

In case of contamination with the highly hydrophilic Paraquat, more than 77.6% of the substance was still present at the application area after cleaning, regardless of the decontamination method. However, the eluted water contained significantly more Paraquat (23.1%) than the sponge (15.5%; *p* < 0.001) and Easyderm® (16.3%; *p* < 0.001) after the cleaning process. The mean CRR_M_ values of sponge and Easyderm® were not significantly different.

Contaminant recovery rates in the skin areas 1, 2 and 5 cm (data not shown in Fig. 3) ranged from < 1% (lower limit of quantification,LLOQ) to 6% for each substance and did not differ significantly between the decontamination methods.

### Efficacy of Easyderm® wiping, twisting or pressing in removing Capsaicin from skin

**Figure 4:**
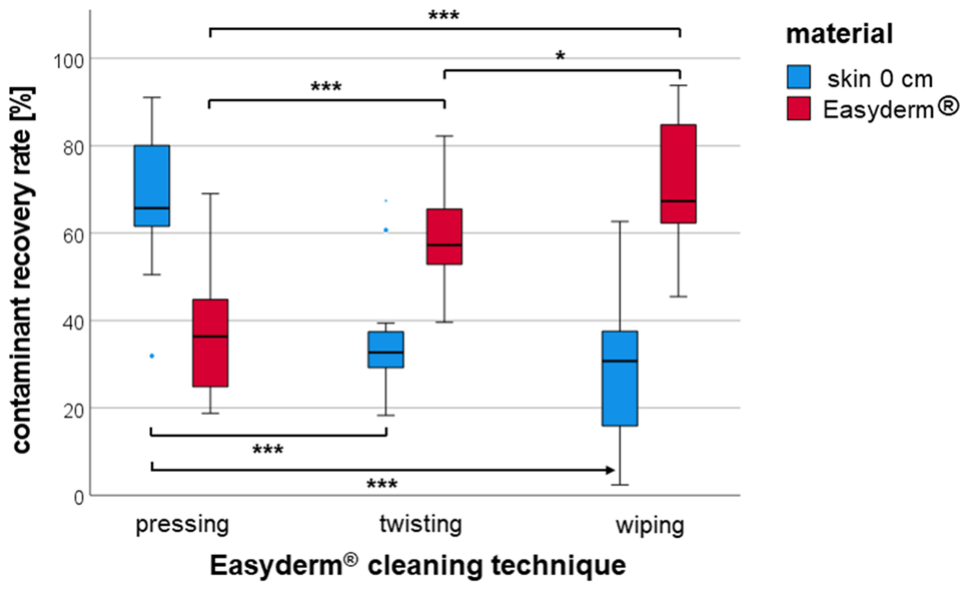
Recovery rates of Capsaicin in skin and Easyderm® after decontamination via Easyderm® pressing, twisting, or wiping. Significant differences are indicated with * (*p* < 0.05), ** (*p* < 0.01) and *** (*p* < 0.001). Non-significant differences are not indicated.

In this part of the study, three different cleaning techniques using the EasyDerm® (pressing, twisting, wiping) were compared in terms of their ability to remove Capsaicin from skin (Fig. 4). The number of replicates was *n* = 20 for each of the three cleaning techniques.

To determine the efficacy of each decontamination method, the CRR of Capsaicin in the application area (CRR_S0_) and in the Easyderm® (CRR_M_) used were analyzed.

Pressing the Easyderm® onto the application field was associated with the highest CRR_S0_ in this skin area (68.0%), which was significantly higher than the twisting (34.8%; *p* < 0.001) and the wiping methods (22.7%; *p* < 0.001). The wiping method was not significantly more effective than the twisting method.

Those findings were consistent with the results of the analysis of the CRR_M_ in the Easyderm® used for each technique. The lowest amount of Capsaicin was detected in the Easyderm® which was only pressed onto the application area (36.8%), while higher contaminant amounts could be found in the twisted (59.4%; *p* < 0.001) and wiped (70.2%; *p* < 0.001) Easyderm®. The difference between the wiped and the twisted Easyderm® was statistically significant as well (*p* < 0.05).

### Impact of exposure time before decontamination of Capsaicin from skin by Easyderm® wiping

**Figure 5:**
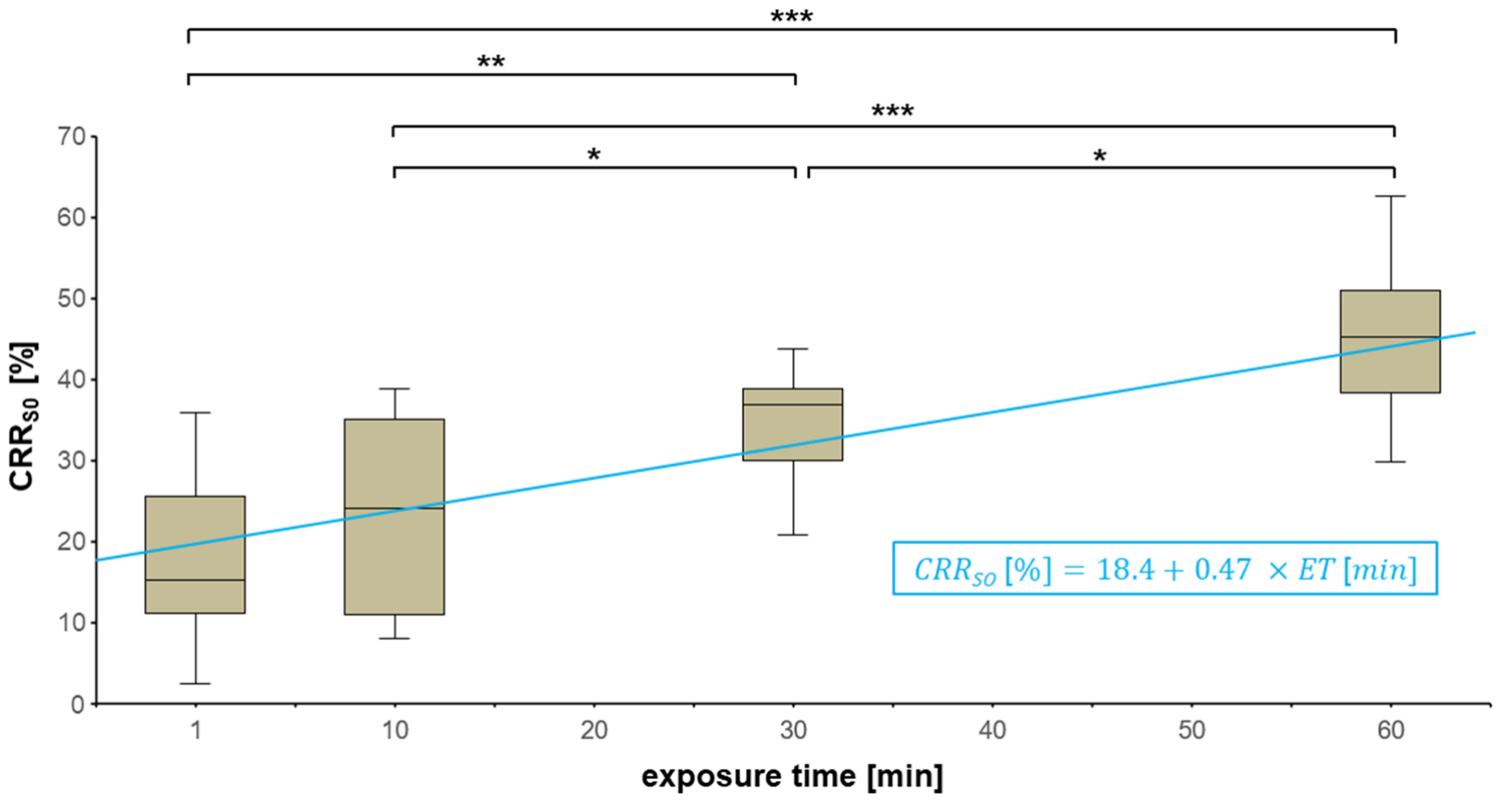
Comparison of different exposure times of Capsaicin to skin before decontamination via Easyderm® wiping method. For better visualization, the abscissa is scaled metrically. Significant differences are indicated with * (*p* < 0.05), ** (*p* < 0.01) and *** (*p* < 0.001). Non-significant differences are not indicated. The regression line calculated via linear regression analysis is drawn in blue. The corresponding recession equation is indicated inside the blue box.

In the third part of the study, the recovery rates of Capsaicin at the application site were examined after exposing the skin to the contaminant for different time spans (1, 10, 30, and 60 min) and then cleaning it by using the Easyderm® wiping method (Fig. 5). The number of replicates was *n* = 10 for the 1, 30 and 60 min time spans each and *n* = 20 for the 10 min time span.

No significant differences in CRR_S0_ were observed between 1 (mean CRR_S0_ = 18.2%) and 10 (mean CRR_S0_ = 22.9%) minutes of exposure time. However, when skin was exposed to Capsaicin for only 1 min, the mean CRR_S0_ was significantly lower than for exposure times of 30 min (mean CRR_S0_ = 34.6%; *p* < 0.01) and 60 min (mean CRR_S0_ = 45.6%; *p* < 0.001). The same could be detected for comparison of 10 min to 30 (*p* < 0.05) and 60 (*p* < 0.001) minutes. Furthermore, a significantly higher amount of Capsaicin was found on the skin after decontamination when exposure time was 60 min compared to an exposure time of 30 min (*p* < 0.05).

Performing a linear regression analysis with CRR_S0_ as dependent and exposure time (ET) as independent variables showed that significantly more Capsaicin could be found at the application area, the higher the exposure time before decontamination (F(1, 48) = 50.1; *p* < 0.001). Both the regression coefficient of ET (t = 7.08; *p* < 0.001) and the constant (t = 9.08; *p* < 0.001) were significant as well. The corresponding regression line was:

$$CRR_{S0} { }\left[ {\text{ \% }} \right] = 18.4 + 0.47{ } \times ET{ }\left[ {{\text{min}}} \right]{ }$$

Additionally, Cohen’s *f*^2^ test was performed to examine the significance of this regression analysis:

$$f^{2} = { }\frac{{R^{2} }}{{1 - { }R^{2} }} = { }\frac{0.500}{{1 - { }0.500}} = 1$$

## Discussion

Rinsing contaminated skin with water (and soap) has been the approach of most emergency medical services in the past. This method has been suggested by many authors^23–26^, because both substances are highly available and have shown adequate removal rates in vitro for several substances, such as glyphosate, phenol and azodrine^27–29^. However, due to increasing evidence of a wash-in effect for contaminants when decontamination is performed with water^30,31^, the search for alternative decontamination methods is an important issue in the context of chemical accidents.

The efficacy of different decontamination methods has been the subject of several studies in the past. Researchers have mainly focused on the comparison of “wet” vs. “dry” decontamination methods: Kassouf et al. demonstrated that dry decontamination with a wiping paper was significantly more effective than conventional rinsing with water and a detergent in removing methyl salicylate, phorate and diethyl malonate from skin^32^. Chilcott et al. in contrast found wet and dry decontamination to be equally effective in removing methyl salicylate from human skin, with wet decontamination providing more consistent results^33^.

In our study, we compared three decontamination methods, two of which we considered as wet decontamination (water rinsing; water-soaked sponge) and one as dry decontamination (Easyderm®).

Our results indicate that the suitability of a decontamination method depends primarily on the chemical properties of the contaminant as well as those of the decontamination material. For example, wiping the contaminated skin with the Easyderm® was far more effective in removing the hydrophobic substance Capsaicin from the application area than either wet decontamination method. This might be expected, as some of the Easyderm®’sactive ingredients are also quite hydrophobic or amphiphilic, for example phospholipid EFA and polysorbate 20. However, out of the two wet methods, wiping the skin with a water-soaked sponge was still significantly more effective than the water rinsing method. In our opinion, this could be due to the mechanical component in form of friction between sponge and skin, which may enhance the effectiveness in removing the contaminant.

The same conclusions can be drawn from the similar, though not as clear, results of decontamination with the only slightly hydrophilic DCEE. Again, dry decontamination achieved the best cleaning results and was significantly more effective than the water rinsing method, but not the sponge method. However, this again might be explainable by the additional mechanical component of the sponge method.

Comparison of the efficacy of wet versus dry decontamination in case of contamination with the very hydrophilic Paraquat again supports our conclusion. Although the CRR_S0_ differences were not significant, the eluted water contained significantly more Paraquat than both sponge and Easyderm®, indicating that in this case wet decontamination was slightly but significantly more effective than the dry method.

In case of contamination with Bromadiolone, the second hydrophobic substance we had used, contaminant recovery rates did not differ significantly between the wet and dry decontamination techniques. However, significantly more Bromadiolone was detected in the eluted water than in the sponge or Easyderm®. Although these differences were not as distinct as for Capsaicin, the Bromadiolone results are not consistent with those of DCEE, Paraquat, and especially Capsaicin. This leads us to the assumption that chemical properties may not determine the most appropriate decontamination method in every case. This in turn could be considered reflective of the partly contradictory study situation on this topic.

In summary, dry decontamination with the Easyderm® (carrying amphiphilic substances) was more effective than wet decontamination methods in removing the more hydrophobic or only slightly hydrophilic substances Capsaicin and DCEE from porcine skin. This observation was consistent with the findings of Kassouf et al., who also found dry decontamination to be more effective when the contaminant was rather hydrophobic^32^. In contrast, wet decontamination was significantly more effective in removing the hydrophilic substance Paraquat. This was similar to the results of Zhai et al., who also found the water rinsing method to be highly suitable for the very water soluble herbicide glyphosate^27^.The suitability of wet decontamination methods however should not only be assessed by the physicochemical properties of the contaminant, but also be evaluated regarding the environmental context of the accident. While rinsing the skin of a patient with water may be most effective for the removal of a hydrophilic substance, this could cause serious hypothermia in case of low ambient temperatures and therefore would rather harm the victim than help him.

In general, we would like to propose shifting the focus to evaluating the physicochemical properties of contaminants and decontamination materials rather than just comparing “wet” versus “dry” methods. We have classified the Easyderm® method as “dry” decontamination and the sponge and water methods as “wet” decontamination in order to place our results in the context of existing literature, where this classification is the most common. The Easyderm®’s active ingredients (aloe barbadensis leaf extract; phospholipid EFA; allantoin; polysorbate 20; aqua; benzalkonium chloride) account for its’ amphiphilic properties, which in our opinion enhance its’ decontamination efficacy. However, this is not a truly “dry” decontamination method due to the certain degree of moisture/liquids it has because of those ingredients. Some authors in the recent years have found dry decontamination to be as or even more effective as wet decontamination in many cases but did not find differences in dry decontamination efficacy between contaminants with different physicochemical properties^32,34^. This however might be due to the fact that the materials they used for dry decontamination did not contain any hydrophilic or lipophilic substances, while in our study, the Easyderm® with its’ amphiphilic properties was significantly more effective in removing hydrophobic than hydrophilic substances from porcine skin.

To our knowledge there are few data on possible spreading of the contaminating substance on the skin when using dry decontamination/wiping techniques. We therefore compared three different motions when using the Easyderm®: pressing, twisting and wiping. Capsaicin was the contaminant of choice for this part of the study, as the Easyderm® wiping technique had already proven to be very effective in removing this substance. The wiping and twisting techniques were significantly more effective for removing Capsaicin than simply pressing the Easyderm® onto the application area. This again supports our impression that the mechanical component in form of friction between cleaning matrix and skin significantly enhances the efficacy of the decontamination method. Furthermore, Wester et al. report that an occlusion of the contaminated skin, which in our case would be similar to the Easyderm® pressing method, may even enhance the risk of absorption of the contaminant into the skin ^29^. While some authors have suggested in the past, that wiping or rubbing may even enhance the dermal penetration of a contaminant^35,36^, we did not observe this effect. The Easyderm® wiping method accounted for the least contaminant recovery rates in the application area out of the three techniques. Spreading of Capsaicin into other parts of the skin caused by Easyderm® wiping led to CRR_S1_ of LLOQ to 6% in “skin 1–5 cm” (data not shown) and was therefore considered as neglectable.

As several authors have stated before, there seems to be an inverse correlation between the exposure time of skin to a contaminant and the efficacy of the following decontamination, regardless of the cleaning method^27,37,38^. Zhai et al. have demonstrated, that rinsing glyphosate-exposed human skin with tap water resulted in approx. 10% less removal when exposure time was 30 min compared to a 3 min-lasting exposure. Similar results have been reported by Loke et al. for exposure of human skin to diethylmalonate ^38^.

The linear regression analysis we performed for cleaning skin, which had been exposed to Capsaicin for four different time spans (1, 10, 30, 60 min), was consistent with those findings. We observed that the efficacy of the Easyderm® wiping method decreased by 0.47 percentage points with every minute of exposure. This should be of particular interest in the context of an actual emergency situation outside of the in vitro laboratory or clinical setting: Emergency services in Germany in 2016/17 had a mean time of arrival at an emergency site of 12.3 min, while the mean time until arrival at the emergency department averaged approx. 47.7 min^39^. According to our linear regression analysis, removing Capsaicin after an exposure time of 12.3 min would have led to a CRR_S0_ of only 24.2%, whereas exposure for 47.7 min would have resulted in 40.8% of the Capsaicin still being detectable in the application area after decontamination. This calculation again emphasizes the importance of the emergency medical services being equipped with effective decontamination materials and techniques: If sufficient decontamination would be performed by the EMS immediately upon arrival, approx. 16.6% more of the contaminating substance could be removed from the patients’ skin compared to decontamination happening not until arrival at the emergency department.

### Limitations

There are slight differences between the natures of human versus porcine skin (vascularization, number/type of perspiratory glands, pH)^40–42^. However, several studies have shown that the transdermal absorption properties of human and porcine skin can be considered to be sufficiently consistent when a substance is applied topically^43–45^. It cannot be entirely excluded that the characteristics of the skin samples might have been altered by the storage temperature of − 20 °C. There is evidence that transdermal penetration of substances varies depending on the site of application^46,47^ as well as between individuals (age, gender, ethnicity, skin type, body mass index, lifestyle)^48^. This could be a potential confounding factor in our study because the porcine skin we used was taken from the back or belly of the pig. It should be noted that non-perfused skin may not respond to the topical application of substances in quite the same way as perfused skin at 37 °C.

In Germany, a minimum flow of 30 l/min is considered as the standard for emergency showers in laboratories^49^. Considering an adults’ average body surface area of 1.91 m^2^ (males)/ 1.71 m^2^ (females)^50^ with a hypothetical contamination of 5%, this would equal a flow of 31.5 ml/cm^2^ (males) – 35.1 ml/cm^2^ (females) per minute. Therefore, we considered our water flow of 24 ml/min (12 ml for 30 s) as appropriate for the contaminated pigskin area of 1 cm^2^*.* However, there is no robust data regarding the amount of water that is typically applied to the victim’s contaminated skin by the emergency services in case of a chemical accident outside of a more or less “controlled” setting like a laboratory. Subsequently, we don’t know to which extent our water rinsing model is comparable to this type of emergency situation. However, rinsing the skin with 50 mL instead of only 12 mL of water did not turn out to be more effective for cleaning the skin in our laboratory model (data not shown).

The choice of model contaminants is mainly based on their physicochemical properties, detectability and broad range of usage, not on the real risk for the population to be exposed to those substances in the applied matrices.

## Conclusions

The suitability of a decontamination method is determined primarily, although not in every case, by the physicochemical properties of the contaminant. The mechanical component in form of friction between skin and cleaning material can increase the efficacy of decontamination. Rapid initiation of the decontamination process is key to reducing skin exposure to contaminants.

Materials that can remove both hydrophilic and hydrophobic contaminants should be available to emergency responders.

## Data Availability

The data presented in this work are available on request from the corresponding author.

## Abbreviations

BRO: Bromadiolone
CAP: Capsaicin
CRR: Contaminant recovery rate
CRR_S0_: Contaminant recovery rate from “skin 0 cm”
CRR_S1_: Contaminant recovery rate from “skin 1–5 cm”
CRR_M_: Contaminant recovery rate from decontamination matrix
DCEE: 2,2’-Dichlorodiethylether
DEP: Diethyl Paraquat
EMS: Emergency medical service
ET: Exposure time
FID: Flame ionization detector
GC: Gas chromatography
HPLC: High performance liquid chromatography
N_2_: Nitrogen
OC: Oleoresin Capsicum
PARA: Paraquat
PDC: Paraquat dichloride
PDM: Paraquat dimethyl sulphate
PT: Prothrombin time
SM: Sulfur mustard

## References

[1] Chemical incidents. Available online: https://www.who.int/health-topics/chemical-incidents/#tab=tab_1. Accessed 21 Oct 2022.

[2] VCI Online. Transport von Chemikalien. Available online: https://www.vci.de/themen/logistik-verkehr/transportsicherheit/transport-von-chemikalien-in-der-chemischen-industrie-wie-viele-chemikalien-werden-transportiert-und-auf-welchen-wegen.jsp. Accessed 15 Dec 2022.

[3] Oracle BI Interactive Dashboards—10 Year Incident Summary Reports. Available online: https://portal.phmsa.dot.gov/analytics/saw.dll?Portalpages&PortalPath=%2Fshared%2FPublic%20Website%20Pages%2F_portal%2F10%20Year%20Incident%20Summary%20Reports. Accessed 21 Oct 2022.

[4] Registered substances—ECHA. Available online: https://echa.europa.eu/information-on-chemicals/registered-substances. Accessed on 15 Dec 2022.

[5] PubChem. Bis(2-chloroethyl) ether. Available online: https://pubchem.ncbi.nlm.nih.gov/compound/8115#section=Use-and-Manufacturing. Accessed 15 Dec 2022.

[6] Dubey V, Gupta AK, Maiti SN, Rao NBSN (2000). Diffusion and sorption of sulfur mustard and bis(2-chloroethyl)ether in elastomers: A comparative study. J. Appl. Polym. Sci..

[7] Dubey V, Rao NBSN, Maiti SN, Gupta AK (1998). Sorption of sulfur mustard and its oxygen analog in black and nonblack-filled butyl rubber membranes. J. Appl. Polym. Sci..

[8] Al Othman ZA, Ahmed YBH, Habila MA, Ghafar AA (2011). Determination of capsaicin and dihydrocapsaicin in Capsicum fruit samples using high performance liquid chromatography. Molecules.

[9] Capsaicin Technical Fact Sheet. Available online: http://npic.orst.edu/factsheets/archive/Capsaicintech.html. Accessed 21 Oct 2022.

[10] Seipelt, I. Capsaicin. *Vidal MMI Germany GmbH,* October 30, 2019. Available online: https://www.gelbe-liste.de/wirkstoffe/Capsaicin_15268#Pharmakokinetik. Accessed 21 Oct 2022.

[11] PubChem. Paraquat. Available online: https://pubchem.ncbi.nlm.nih.gov/compound/15939. Accessed 15 Dec 2022.

[12] Sante, D. EU Pesticides Database (v.2.2) Active substance. Available online: https://ec.europa.eu/food/plant/pesticides/eu-pesticides-database/active-substances/?event=as.details&as_id=952. Accessed 21 Oct 2022.

[13] Botella R, Sastrf A, Castells A (1985). Contact dermatitis to Paraquat. Contact Dermat..

[14] Tungsanga K, Chusilp S, Israsena S, Sitprija V (1983). Paraquat poisoning: Evidence of systemic toxicity after dermal exposure. Postgrad. Med. J..

[15] PubChem. Bromadiolone. Available online: https://pubchem.ncbi.nlm.nih.gov/compound/54680085#section=Flash-Point. Accessed 15 Dec 2022.

[16] Simmchem. Aufnahme des Wirkstoffs Bromadiolon in Anhang I der Biozidrichtlinie—Simmchem. Available online: https://simmchem.com/2009/aufnahme-des-wirkstoffs-bromadiolon-in-anhang-i-der-biozidrichtlinie/. Accessed 15 Dec 2022.

[17] Umweltbundesamt. Rodentizide. Available online: https://www.umweltbundesamt.de/themen/chemikalien/biozide/biozidprodukte/rodentizide. Accessed 21 Oct 2022.

[18] Kruse JA, Carlson RW (1992). Fatal rodenticide poisoning with brodifacoum. Ann. Emerg. Med..

[19] Swigar ME, Clemow LP, Saidi P, Kim HC (1990). “Superwarfarin” ingestion. Gen. Hosp. Psychiatry.

[20] Rutović S, Dikanović M, Mirković I, Lojen G, Marcikić M (2013). Intracerebellar hemorrhage caused by superwarfarin poisoning. Neurol. Sci..

[21] Zuo W, Zhang X, Chang J-B, Ma W-B, Wei J-J (2019). Bromadiolone poisoning leading to subarachnoid haemorrhage: A case report and review of the literature. J. Clin. Pharm. Ther..

[22] Narlı ÖZ (2016). Tekrarlayan Mesleksel Deri Maruziyetine Bağlı Süpervarfarin Zehirlenmesi Gelişen Bir İşçi Olgusu. Turk. J. Haematol..

[23] Levitin HW (2003). Decontamination of mass casualties–re-evaluating existing dogma. Prehosp. Disaster Med..

[24] Brennan RJ, Waeckerle JF, Sharp TW, Lillibridge SR (1999). Chemical warfare agents: Emergency medical and emergency public health issues. Ann. Emerg. Med..

[25] ASPR TRACIE. OSHA Best Practices for Hospital-Based First Receivers of Victims from Mass Casualty Incidents Involving the Release of Hazardous Substances | Technical Resources. Available online: https://asprtracie.hhs.gov/technical-resources/resource/1062/osha-best-practices-for-hospital-based-first-receivers-of-victims-from-mass-casualty-incidents-involving-the-release-of-hazardous-substances. Accessed 25 Oct 2022.

[26] Bromberg BE, Song IC, Walden RH (1965). Hydrotherapy of chemical burns. Plast. Reconstr. Surg..

[27] Zhai H, Chan HP, Hui X, Maibach HI (2008). Skin decontamination of glyphosate from human skin in vitro. Food Chem. Toxicol..

[28] Pullin TG, Pinkerton MN, Johnston RV, Kilian DJ (1978). Decontamination of the skin of swine following phenol exposure: A comparison of the relative efficacy of water versus polyethylene glycol/industrial methylated spirits. Toxicol. Appl. Pharmacol..

[29] Wester RC, Maibach HI (1985). In vivo percutaneous absorption and decontamination of pesticides in humans. J. Toxicol. Environ. Health.

[30] Chan HP, Zhai H, Hui X, Maibach HI (2013). Skin decontamination: Principles and perspectives. Toxicol. Ind. Health.

[31] Moody RP, Maibach HI (2006). Skin decontamination: Importance of the wash-in effect. Food Chem. Toxicol..

[32] Kassouf N, Syed S, Larner J, Amlôt R, Chilcott RP (2017). Evaluation of absorbent materials for use as ad hoc dry decontaminants during mass casualty incidents as part of the UK’s Initial Operational Response (IOR). PLOS ONE.

[33] Chilcott RP, Mitchell H, Matar H (2019). Optimization of nonambulant mass casualty decontamination protocols as part of an initial or specialist operational response to chemical incidents. Prehosp. Emerg. Care.

[34] Thors L (2023). Comparison of skin decontamination strategies in the initial operational response following chemical exposures. Toxicol. Vitro.

[35] Hasler-Nguyen N, Fotopoulos G (2012). Effect of rubbing on the in vitro skin permeation of diclofenac-diethylamine 1.16% gel. BMC Res. Notes.

[36] Ishii H, Todo H, Sugibayashi K (2010). Effect of sebum and ointment rubbing on the skin permeation of triamcinolone acetonide from white petrolatum ointment. Biol. Pharm. Bull..

[37] Fenske RA, Schulter C, Lu C, Allen EH (1998). Incomplete removal of the pesticide captan from skin by standard handwash exposure assessment procedures. Bull. Environ. Contam. Toxicol..

[38] Loke W-K (1999). Wet decontamination-induced stratum corneum hydration—effects on the skin barrier function to diethylmalonate. J. Appl. Toxicol. JAT.

[39] Schmiedel, R. *Leistungen des Rettungsdienstes 2016/17* (2019).

[40] Czembirek H, Freilinger G, Gröger L, Mandl H, Zacherl H (1974). Zur Gefässversorgung der Bauchhaut des Schweines. Acta Anat..

[41] Simon GA, Maibach HI (2000). The pig as an experimental animal model of percutaneous permeation in man: Qualitative and quantitative observations–an overview. Skin Pharmacol. Appl. Skin Physiol..

[42] Meyer W, Neurand K (1991). Comparison of skin pH in domesticated and laboratory mammals. Arch. Dermatol. Res..

[43] Bronaugh RL, Stewart RF, Congdon ER (1982). Methods for in vitro percutaneous absorption studies. II. Animal models for human skin. Toxicol. Appl. Pharmacol..

[44] Qvist MH, Hoeck U, Kreilgaard B, Madsen F, Frokjaer S (2000). Evaluation of Göttingen minipig skin for transdermal in vitro permeation studies. Eur. J. Pharm. Sci..

[45] Schmook FP, Meingassner JG, Billich A (2001). Comparison of human skin or epidermis models with human and animal skin in in-vitro percutaneous absorption. Int. J. Pharm..

[46] Rougier A (1986). Regional variation in percutaneous absorption in man: measurement by the stripping method. Arch. Dermatol. Res..

[47] Feldmann RJ, Maibach HI (1967). Regional variation in percutaneous penetration of 14C cortisol in man. J. Invest. Dermatol..

[48] Dąbrowska AK (2014). The relationship between skin function, barrier properties, and body-dependent factors. Skin Res. Technol..

[49] Working Safely in Laboratories. Available online: https://downloadcenter.bgrci.de/resource/downloadcenter/downloads/DGUV-Information_213-851_Gesamtdokument.pdf. Accessed 23 Apr 2023.

[50] Sacco JJ, Botten J, Macbeth F, Bagust A, Clark P (2010). The average body surface area of adult cancer patients in the UK: a multicentre retrospective study. PLoS ONE.

